# Assessment of malathion toxicity on cytophysiological activity, DNA damage and antioxidant enzymes in root of *Allium cepa* model

**DOI:** 10.1038/s41598-020-57840-y

**Published:** 2020-01-21

**Authors:** Akhileshwar Kumar Srivastava, Divya Singh

**Affiliations:** Laboratory of Cytogenetics, Department of Botany, Banaras Hindu University, Varanasi, 221005 India

**Keywords:** Plant sciences, Plant stress responses, Environmental sciences

## Abstract

The current study was emphasized to assess the effect of malathion on root system (cell division and kinetics of the root elongation) and stress related parameters in *Allium cepa* L. The roots were exposed to different concentrations (0.05, 0.13, 0.26, 0.39 and 0.52 g/L) of malathion for different treatment periods (4, 8 and 18 h). The results revealed that malathion application affected the growth rate and cell division in root tips. The root elongation kinetics were impaired at 0.13 to 0.52 g/L concentrations. Reduction in tissue water content (TWC) indicated the limited osmotic adjustment due to membrane damage. Further, a decrease in sucrose content was observed in contrast to the accumulation of proline (upto 0.39 g/L). Moreover, malathion exposure elevated the levels of lipid peroxidation followed by changes in antioxidant enzymes status. The activities of ascorbate peroxidase (APX) and glutathione reductase (GR) were down-regulated whereas the activities of catalase (CAT), glutathione-S-transferase (GST) and superoxide dismutase (SOD) were up-regulated except in 0.52 g/L malathion. The molecular docking study of malathion with CAT, GST, SOD, APX and GR also supported of above results for their activity. All these physiological responses varied with increasing malathion concentration and duration of treatment. The single cell gel electrophoresis results showed that all concentrations of malathion induced DNA damage in root cells. The findings depicted that malathion application induces cytotoxic and phytotoxic effects mediated through oxidative stress and subsequent injuries.

## Introduction

In modern agricultural system, the different types of pesticides are adopted to control pests, weeds or plant diseases. The implications of pesticides is regarded as highly effective and accepted for to protect the plants against pest attack and has widely contributed to enhance the agricultural production^1–3^. The organophosphorous compounds are sharing the major portion of pesticide consumption in India because they have less effect on food safety, human health and environment; are mainly recognized as non-toxic to plants^4,5^. Malathion is one of the oldest organophosphate pesticides recommended in the 1950’s^6^. It has been extensively used in many countries. In India, it is one of the five most commonly used pesticides to control insects, flies, mosquitoes and animal parasites^7^. Earlier reports show that malathion produces perilous effects on the environment when it is mixed with soil or directly applied to plants^8^. The absorption of malathion by pea plant has been studied by Getenga *et al*. (2000) and it concluded that, the uptake rate of malathion reached maximum after 6 hours of application^9^. Malathion exposure affected physiological activity and yield in potato and *Picea sitchensis*^10,11^. Malathion also alters mitotic activity, chromosomal structure and changes stability of DNA template and expression of proteins^12^.

Although, the mechanism of action of malathion is not well studied, however envisaging that it has similar mechanism of action to methyl parathion. When methyl parathion is sprayed on the crops, it is broken down in the presence of sunlight and converted into a toxic product known as methyl paraoxon. Methyl paraoxon phosphorylates the active site of the enzyme acetylcholinesterase leading to its deactivation and further inhibition of hydrolysis of acetylcholine^13^.

In addition, presence of OPs residue in vegetables and fruits may induce health problems to human^14^. Plants transferring their preserved genetic materials though food chain are frequently imparted to assess the genotoxicity and mutagenicity developed from risk factors. *Allium cepa* is commonly used in laboratories to know the toxicity of chemical compounds and environmental risk factors. It also shows good correlation with other test systems. *Allium* test has an imperative way for differentiating the environmental contamination and their results could be approached for signaling to other test systems^15^. Roots are the most vulnerable and reliable system to study the mechanism of pesticide interaction as a primary receptor. Generally, pesticides are not adsorbed, absorbed or degraded may also affect on non target species. These pesticides may reach into the soil and transported to other parts of plant through roots that may lead to alterations in physiological processes^16^.

Pesticide application leads to overproduction of reactive oxygen species (ROS) resulting lipid peroxidation, cell membrane damage, protein oxidation, enzyme inactivation, DNA and RNA damage. Plants have different strategies to tolerate pesticide induced toxicity. They accumulate an array of metabolites including amino acids and osmoprotectants (compatible solutes) under stressful conditions. Compatible solutes like sucrose, proline, trehalose, polyols and quaternary ammonium compounds assure the plants from stress via various mechanisms e.g. detoxification of ROS, maintain the membrane integrity, osmotic adjustment and providing the stability to proteins/enzymes^17^. Further, plants have established a complex antioxidant system to mitigate and repair damages caused by ROS. Enzymatic antioxidative system has been one of the most imperative mechanisms of plant to counter environmental stresses. In general, toxic organic compounds can alter the function of antioxidant enzymes including ascorbate peroxidase (APX), superoxide dismutase (SOD), catalase (CAT), glutathione reductase (GR) and glutathione S-transferase (GST) that reflect not only the level of toxicity but also the stress tolerance capacity of plants^18^. Now a days, the protein interaction with molecules has become a great tool in the science researches; life sciences^19^, medicine^20^, environment^21^ and chemistry^22^. The molecular docking is a way to address and know the molecular sites, determine the predominant the interaction mode and binding efficiency between the protein and ligand^23^ that give a 3D-crystal structure of the protein-ligand complexes^24^. Similarly, the pesticides like malathion could play major role to disrupt the natural structure of proteins/enzymes by interacting with their residues.

The present study elucidated the effect of malathion at different doses on root development (elongation kinetics and cell division), accumulation of compatible solutes (sucrose and proline), and antioxidant enzymes (CAT, GST, SOD, APX and GR) level along with interactive study of malathion 3D structure.

## Materials and Methods

The healthy bulbs of *A. cepa* L. were purchased from market. For the growth experiment, these bulbs were placed in sand and rooted at room temperature. Then the bulbs having roots of 2–3 cm length were immersed in Hoagland’s nutrient solution having 0.05, 0.13, 0.26, 0.39 and 0.52 g/L malathion for 5 days. Simultaneously, control roots were placed in Hoagland’s nutrient solution^25^. The solutions were renewed after every 24 h. Roots were measured once per day. Root length of control was taken 100% growth. EC_50_, 2 × EC_50_ and ½ × EC_50_ values were calculated by concentration-response curve applying log-logistic functions. EC_50_ value represents the concentration that inhibited root length by 50% in comparison to control.

For cytogenetic and physiological analysis, bulbs were placed in sand at room temperature. After 4 days, seedlings were transferred into Hoagland′s nutrient solution having 0.05, 0.13, 0.26, 0.39 and 0.52 g/L malathion for 4, 8 and 18 h. Cytogenetical studies were carried out by fixing roots in ethanol and glacial acetic acid (3:1 v/v) for 24 h, followed by hydrolysis in 1 N HCl at 60 °C for 5 minutes. Then root tips (1 mm) were cleaned and stained with 2% aceto-carmine^26^. The mitotic index (MI) in percentage was calculated by counting the cells of mitotic phases in total number of cells (1000 cells/slide).

For determination of tissue water content (TWC), fresh weight (FW) of the exposed roots was recorded at the end of each treatment period. Although, dry weight (DW) was determined after drying the roots at 60 °C in the oven for 48 h. The tissue water content (TWC) of the roots was calculated and expressed as percentage.

Sucrose content was measured according to Van Handel (1968) using anthrone reagent. Fresh roots (100 mg) were homogenized in 80% ethanol and centrifuged at 5,000 × g for 15 min^27^. The reducing sugars were destroyed by placing reaction tubes in boiling water bath (10 min). After cooling, 30% aqueous KOH was added and kept at 100 °C (10 min). When tubes were cooled, anthrone reagent was added in it and incubated at 38 °C (20 min) and absorbance was recorded at 620 nm using sucrose (0.1 mg/mL) as standard. The sucrose content was presented in µg g^−1^ FW.

Free proline content was accounted by the modified method of Bates *et al*.^28^. The roots (500 mg) were homogenized in ice cold sulphosalicylic acid (3%). After centrifuging at 5,000 × g for 15 min (4 °C), about 2 mL extract was blended with glacial acetic acid (2 mL) and ninhydrin reagents (2 mL). Then reaction mixture was incubated in water bath (boiling) for 30 min. After cooling, 4 mL of toluene was added. The absorbance was recorded at 520 nm. The amount of proline was presented in µg g^−1^ FW.

The extent of lipid peroxidation was determined by measuring malondialdehyde (MDA) concentration^29^. Fresh roots (600 mg) were homogenized in 3 mL reaction mixture containing 20% trichloroacetic acid with 0.5% thiobarbituric acid. The homogenates were heated at 95 °C (30 min). After ice cooling, samples were centrifuged at 10,000 × g (10 min). The indifferent absorbance at 600 nm was substracted for turbidity correction. The MDA concentration was presented in nmol g^−1^ FW.

For examination of antioxidant enzyme activities, about 1 g fresh roots were homogenized in 2 mL of phosphate buffer (0.05 M, pH 7.0) followed by centrifugation at 15,000 × g for 20 min (4 °C). The supernatant was collected to estimate the enzyme activities. Catalase (CAT) activity was estimated to monitor the decline in absorbance at 240 nm in the reaction mixture of phosphate buffer (0.05 M, pH 7.0) and H_2_O_2_ (10 mM). The enzyme activity was presented in mM of H_2_O_2_ utilized min^−1^ g^−1^ FW^30^. Glutathione-S-transferase (GST) activity was determined in a reaction mixture of phosphate buffer (0.05 M, pH 7.0), reduced glutathione (10 mM) and 1-chloro-2,4-dinitrobenzene (10 mM). The absorbance was recorded at 340 nm and enzyme activity was expressed as μmol min^−1^ g^−1^ FW^31^. Superoxide dismutase (SOD) activity was quantified^32^ in a reaction mixture (3 mL) of 0.05 M phosphate buffer (pH 7.8), 3 mM EDTA, 2.25 mM nitroblue tetrazolium chloride (NBT), 0.2 M methionine, 1.5 M sodium carbonate and 2 µM riboflavin. The reaction was started by placing tubes under 40 W fluorescent tube for 10 min, and stopped in dark condition. The absorbance was measured at 560 nm. The enzyme activity was also considered as enzyme required to inhibit 50% NBT photoreduction and presented as unit g^−1^ FW. Ascorbate peroxidase (APX) was assayed^33^ in a reaction mixture (3 mL) containing phosphate buffer (0.05 M, pH 7.0), ascorbic acid (0.5 mM), EDTA (0.2 mM) and H_2_O_2_ (10 mM). The absorbance of reaction mixture was recorded at 290 nm for 3 minutes and enzyme activity was represented as μmol ascorbate oxidized min^−1^ g^−1^ FW. Glutathione reducatase (GR) activity was estimated^34^ in a reaction mixture of 2 mL containing 0.05 M potassium phosphate buffer (pH 7.0), 0.1 mM nicotinamide adenine dinucleotide phosphate (NADPH), 3 mM EDTA and 1 M oxidized glutathione (GSSG). The absorbance was taken at 340 nm and enzyme activity was presented in µmol NADPH min^−1^ g^−1^ FW.

Molecular docking was performed on antioxidant enzymes: catalase (PDB:ID 5gkn)^35^, glutathione-S-transferase (PDB:ID 1gnw)^36^, superdioxide dismutase (PDB:ID 1ba9)^37^, ascorbate peroxidase (PDB:ID 2xi6)^38^ and glutathione reductase (PDB:ID 2hqm)^39^ with malathion. The 3D structure of malathion (PubChem ID: CID_4004) was retrieved from the PubChem online data server^40^. PatchDock web server was adopted for analysis of molecular interaction between ligand and receptor. The thousands solutions with score, area, and six-dimensional transformation space were obtained from Patch-Dock, further it was transferred into FireDock web server to obtain the ten best solutions based on global energy^41^. The obtained solutions from the FireDock was ranked on the basis of minimum global binding energy. The Discovery Studio 4.5 Client was used to visualize the crystal structure of complexes^42^.

For alkaline single cell gel electrophoresis^43^, roots were placed in petri plate kept on ice. The roots were immediately chopped with a fresh razor blade in 400 µL of cold Tris-buffer (0.4 M, pH 7.5). Then a 1:1 mixture of nuclear suspension and 1% low melting point agarose (LMPA) in phosphate-buffered saline were coated on the pre-coated slides with 1% normal melting point agarose (NMPA) at 40 °C with cover slip. After completion of gelling step of LMPA, cover slip was gently removed. The slides having LMPA-embedded nuclei were transferred in a horizontal gel electrophoresis tank having fresh and chilled electrophoresis buffer (0.3 M NaOH and 1 mM EDTA, pH > 13) for 15 min followed by electrophoresis at 0.7 V cm^−1^ (20 V and 0.3 A) for 20 min at 4 °C. Slides were rinsed 3 times with distilled water and neutralized with Tris buffer (0.4 M Tris, pH 7.5). After immersion in cold distilled water for 5 min., nuclei were stained with 80 µL of ethidium bromide (20 µg mL^−1^) for 5 min. The slides were rinsed with cold distilled water to remove excess ethidium bromide and covered with a cover slip. All these steps were carried under dim light to protect the DNA damage. Analysis of slides was carried out using fluorescence microscope (Olympus BX 51). Cells with damaged DNA visualized as comets and their tail length, tail DNA (%) and olive moment were assessed by Open Comet software. The cells were classified into five categories based on tail DNA (%): undamaged (<5%), low damage (5–25%), moderate damage (26–45%), high damage (46–80%) and extreme damage (>80%)^44^.

### Data analysis

Root elongation kinetics were elucidated using the Gompertz, logistic, log-logistic and Weibull models, selecting the one based on the best fit in Akaike Information Criterion (AIC) and Bayesian Information Criterion (BIC). The free parameters namely length of lag phase (λ), growth rate (µ) and maximum growth (A) shared by these models were applied as descriptors of root elongation kinetics. The functions of “drc” package^45^ for R programming language^46^ were used to describe root elongation kinetics.

The differences in root elongation kinetics among the concentrations of malathion were evaluated through one-way analysis of variance (ANOVA) and Kruskal-Wallis H tests following TukeyHSD and Dunn post hoc tests. The homoscedasticity or heteroscedasticity of the results were evaluated by Breuch-Pagan test. Further, the differences in MI, TWC, sucrose and proline contents, activities of antioxidant enzyme (CAT, GST, SOD, APX and GR) and DNA damage among the concentrations of malathion were evaluated through ANOVA and Kruskal-Wallis H tests followed by Dunn post hoc test.

One-way analysis of variance (ANOVA), Kruskal-Wallis H tests following TukeyHSD and Dunn post hoc tests were performed with the functions of “stats” and “lmtest”^47^ packages of the R programming language.

## Results and Discussion

**Figure 1:**
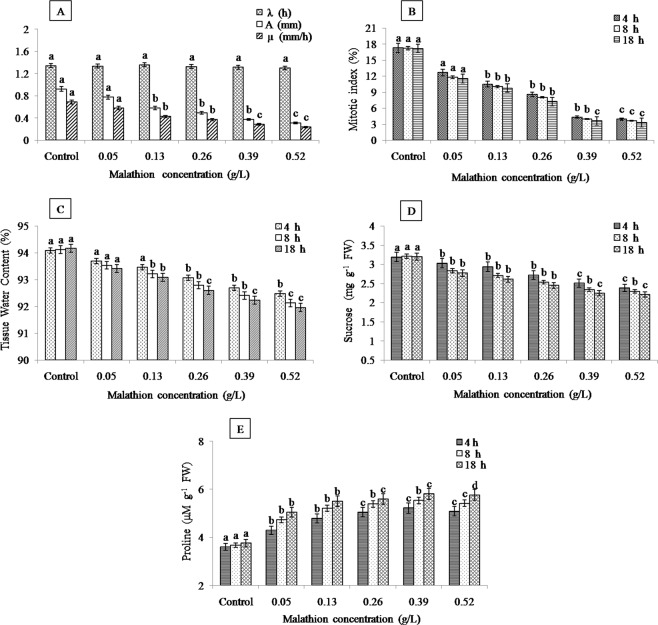
Graphical representation of malathion effect on (**A**) root elongation kinetics, (**B**) mitotic index, (**C**) tissue water content, (**D**) sucrose content and (**E**) proline content in roots of *A. cepa* L. (significant at p ≤ 0.05).

Except λ, the kinetic studies of root elongation emphasized significant decrease in growth rate and final root length. Roots treated with malathion at 0.13, 0.26, 0.39 and 0.52 g/L had significantly lower µ and A in comparison to control (Fig. 1A). The parameters related to root elongation are sensitive endpoints to appraise phytotoxic consequences due to the physical interaction of root with pesticide^16^. The 50% reduction in root length (EC_50_) was observed at 0.26 g/L, 2 × EC_50_ at 0.52 g/L and ½ × EC_50_ at 0.13 g/L. The differences observed in root elongation parameters were also observed in case of MI that was gradually reduced with the escalated concentration and treatment time (Fig. 1B). The observed MI of roots was lower at 0.13, 0.26, 0.39 and 0.52 g/L malathion treatment in comparison to control. The inhibition based on concentration for MI described the cytotoxicity of malathion in *A. cepa* L. Similar effects on mitotic activities were reported in many studies^12,48,49^ and it has been attributed by the interference of insecticides in the normal cell cycle leading to decrease in the number of dividing cells.

The plant water status under any stress condition has predominant role in its survival and growth. Dose and time dependent reduction in TWC of malathion-stressed roots was observed in the present study (Fig. 1C) which indicated the limited osmotic adjustment and it also related with biomass control^50^. This event may happen from the membrane damage causing leakage of water as a result of change in permeability of membrane^51^. The similar outcomes were obtained by Singh *et al*.^52^ in soybean; Gafar *et al*. in cucumber following different chemical treatment^53^. Our results are consistent with those of Nasrabadi and Dhumal who reported malathion-induced reduction in water content, FW, DW of tomato and brinjal leaves^51^.

The sucrose content also decreased significantly in the roots with the escalated malathion concentration and exposure time (Fig. 1D). Changes in sucrose contents as part of carbohydrates are of specific significance because of its direct association with physiological processes such as photosynthesis, translocation and respiration^54^. The severely reduced sucrose levels was recorded at 0.52 g/L (18 h) might be the trade-off for homeostasis. It has been reported that the consumption of carbohydrates during the stress condition was critical for plant survival^55^. Prensser *et al*. reported that the malathion implementation in *Vicia faba* plants lowered the soluble sugar content^56^. Application of diuron herbicide decreased soluble carbohydrates in wheat^57^ while an increase in those soluble carbohydrates was noticed in soybean plants^58^. In our case possibly, pesticides arrest the germination and growth of plants by degradation of carbohydrates reserves during germination and growth^59^. It can also be suggested that sugar metabolism is very sensitive to changes in higher stress intensity.

Earlier, the many scientific research reported about the changes in proline content in different plant tissues. In this study significant increase in the proline accumulation was recorded with increasing malathion concentration upto 0.39 g/L in malathion stressed plants after which reduction was observed (Fig. 1E). The decrease in proline content reflects sensitive nature of plant at higher doses of malathion. The increased or decreased level of proline correlated with the magnitude of stress which governs the physiological status, growth and yield of the plants. Whereas accumulation of proline has might have helped the test crop under xenobiotic stress and to maintain the membrane stability, water relations and energy metabolism^60^.

**Figure 2:**
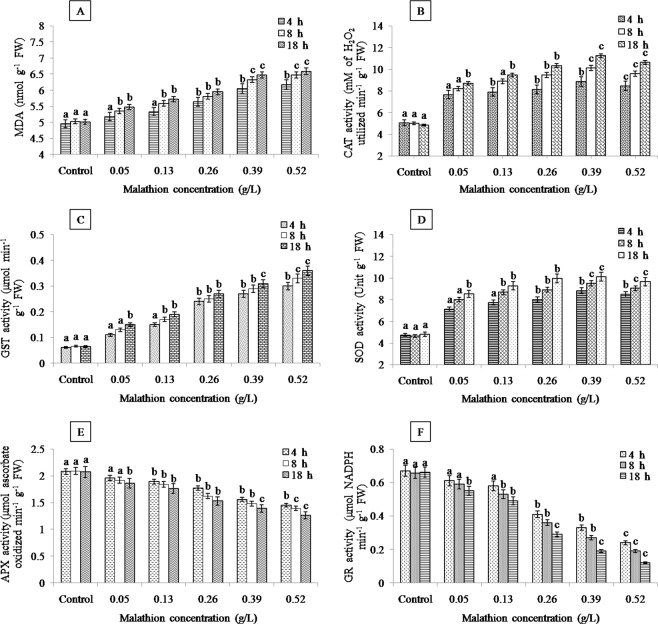
Effects of malathion on (**A**) malondialdehyde (MDA) content, (**B**) catalase (CAT) activity, (**C**) glutathione-S-transferase (GST) activity, (**D**) superoxide dismutase (SOD) activity, (**E**) ascorbate peroxidase (APX) activity and (**F**) glutathione reductase (GR) activity in roots of *A. cepa* L. (significant at p ≤ 0.05).

Over-generation of ROS is a highly sensitive response of plants to environmental factors that aggravates the membrane lipid peroxidation in plants and MDA content is an essential indicator to assess the peroxidation of membrane lipids. Lipid peroxidation could be considered as first step for cellular membrane damage by organophosphates^61^. Malathion (0.05–0.52 g/L) induced significant oxidative damage in roots as indicated by the increased MDA content compared to control root (Fig. 2A). Similarily, upon exposing of *Glycine max* L. to insecticide deltamethrin led for escalation of lipid peroxidation in the leaves and roots^62^. The increased lipid peroxidation in the present study explained that the ROS-induced damage could be one of the main toxic effects of malathion.

To alleviate and repair the damage produced from these ROS, plant have developed antioxidant systems. In this study, the APX and GR activity decreased significantly as malathion treatment increased, indicating the sensitivity of these enzyme (Fig. 2E,F). The activities of CAT, GST and SOD increased upto 0.39 g/L indicating that this optimum concentration may induce for activation of enzymatic ROS scavenging mechanisms (Fig. 2B–D). However, the declination in CAT activity at the highest dose (0.52 g/L) reflected that H_2_O_2_ was not efficiently detoxified by this enzyme. The observed high MDA content in the present work indicated high H_2_O_2_ level that was correlated to the inhibition of CAT activity^63^. Lowering in GST activity at 0.52 g/L concentration indicated the sensitivity of enzyme. Moreover, decline in SOD activity at the highest dose (0.52 g/L) might be associated with inhibition of this enzyme at higher level of ROS^64^. The similar response by some herbicides was also noted in rice^65^, soybean^66^ and wheat^67^. Our results suggest that the reduction in APX and GR activity in roots of *A. cepa* L. signify damage of plant against oxidative stress at high levels of malathion. In consequence, CAT, GST and SOD enzymes have less susceptibility for malathion than APX and GR.

**Figure 3:**
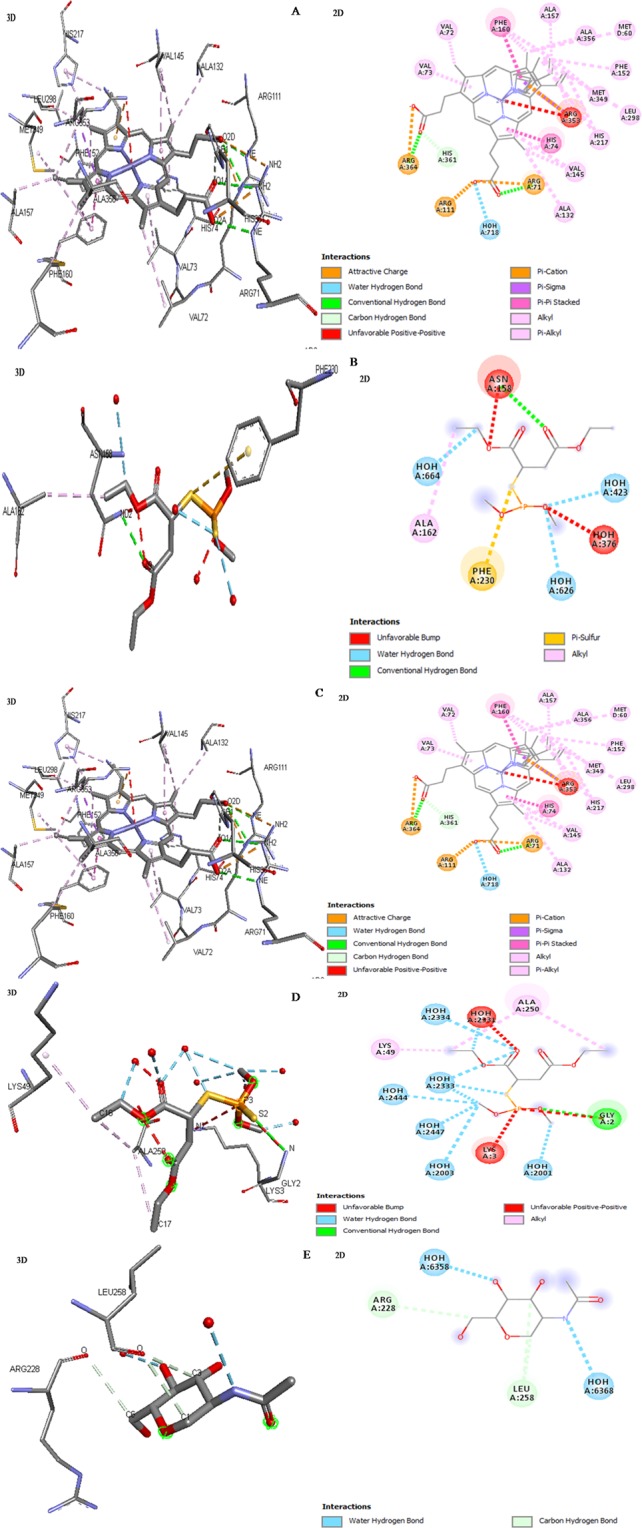
Visualization of malathion in complexation with antioxidant enzymes represents 3D and 2D structures (**A**) catalase (CAT), (**B**) glutathione-S-transferase (GST), (**C**) superoxide dismutase (SOD), (**D**) ascorbate peroxidase (APX) and (**E**) glutathione reductase (GR).

Further, we performed molecular docking study to reveal the interactions between malathion and antioxidant enzymes (Fig. 3). The possible confirmations of complexes was calculated by using FireDock online server. The crystal structures (2D/3D) of malathion and antioxidant enzymes complexation shows the involvement of many interactions. The H-bond interactions make strong complexes between malathion and antioxidant enzymes.

In malathion-CAT complex, the atoms NE (ARG364) and NH2 (ARG71) of catalase contributed to make H-bond interactions with O2A and O2D of malathion compound, respectively (Fig. 3A). The complex of malathion-gluthion-S-transferase showed that the atoms (N1) of residue (GLU66) and N of SER67 made H-bond interactions with OE2 and O11 atoms of malathion, respectively, other atoms (N and O) of residue (VAL54) anchored through H-bond interactions to atoms O2 and N2 of malathion (Fig. 3B). The structural complex of malathion-SOD exhibited that the interactive site OE2 (GLU121) and N (ARG143) attaches to C18 and O5 of malathion, respectively, by H-bond (Fig. 3C). The malathion exhibited H-bond interaction with N atom of crucial residue (GLY2) showing the active site of ascorbate peroxidase (Fig. 3D). A single atom (O) of residue LEU258 of gluthion-reductase displayed the strong H-bond interactions with two atoms (C1 and C3) of malathion (Fig. 3E).

The global binding energy and H-bond was found attributed to strong attachment of malathion and antioxidant enzymes (Table 1). The binding energy of each complexes was dealing for strong binding and its higher negative value assigns to higher free binding energy and thus stronger interaction possibility^41^. The positive global binding energy of malathion for CAT, GST, and SOD were to be found 31.84, 27.53, and 27.50 kcal/mol respectively, indicating that the enzymes CAT, GST, SOD may be overexpressed under malathion stress (Table 1). The negative binding energy (−14.87 and −11.13 kcal/mol) of malathion for APX and GR suggested that the activity of APX and GR after malathion treatment might be suppressed (Table 1). The H-bond also contributes to global binding energy for strong interaction of malathion with antioxidant enzymes, were −7.37, −6.51, −9.13, −3.72 and −3.10 kcal/mol for CAT, GST, SOD, APX and GR, respectively (Table 1). Overall, the interaction parameters and binding energy obtained from molecular docking showed that the antioxidant enzymes could be highly sensitive against malathion activity in plant system.

**Table 1:** Inherent global binding energy and H-bond attribute the complex formation between malathion and antioxidant enzymes.

Antioxidant enzymes	Global binding energy (kcal/mol)	H-bond kcal/mol
CAT	31.84	−7.37
GST	27.53	−6.51
SOD	27.5	−9.13
APX	−14.87	−3.72
GR	−11.13	−3.1

**Figure 4:**
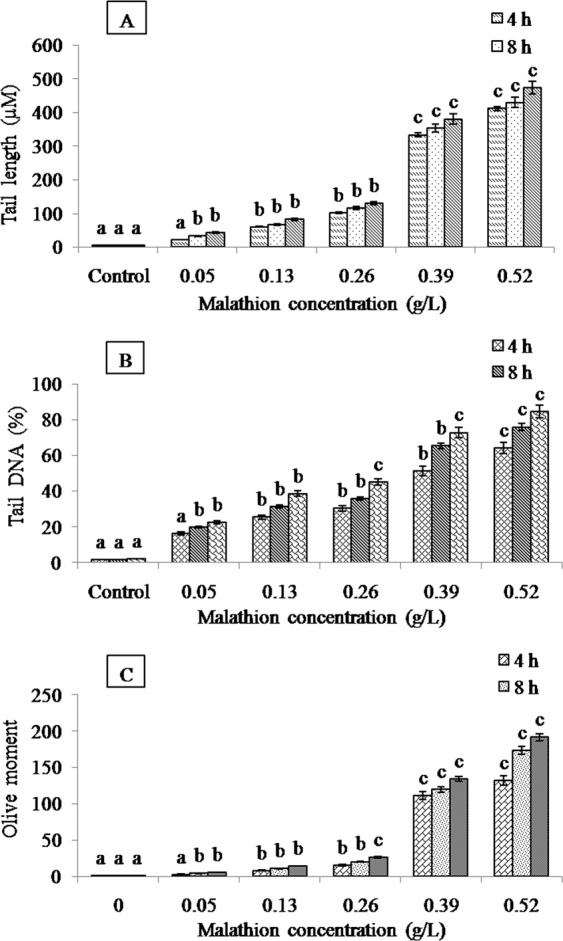
DNA damage in root cells of *A. cepa* L. under different concentrations of malathion at different treatment periods (significant at p ≤ 0.05). Effect of malathion on (**A**) tail length, (**B**) tail DNA and (**C**) olive moment.

**Figure 5:**
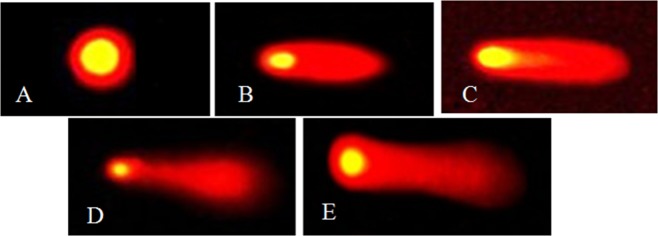
Classification of comets based on tail DNA. Categories of comet: (**A**) undamaged cells (tail DNA% < 5); (**B**) low damaged cells (tail DNA% 5–25); (**C**) moderately damaged cells (tail DNA% 26–45); (**D**) highly damaged cells (tail DNA% 46–80); (**E**) extremely damaged cells (tail DNA% > 80).

At present, single cell gel electrophoresis is extensively used in environmental monitoring and toxicological studies^44^. The genotoxic effects of malathion on roots of *A. cepa* L. was deliberated by using single cell gel electrophoresis test with three scales (tail length, tail DNA% and olive tail moment). The results revealed a concentration and time-dependent increase in all the three parameters (tail length, tail DNA and olive moment) of comet with respect to control (Fig. 4). According to the classification of comets, the DNA damage in cells of control root was considered as undamaged category (Fig. 5A). It was observed low damaged cells in the roots exposed to 0.05 g/L after 4–18 h and 0.13 g/L after 4 h (tail DNA% 5–25, Fig. 5B). The moderately damaged cells appeared in the 0.13 g/L treatment group after 8 and 18 h and in the 0.26 g/L treatment group (tail DNA% 26–45, Fig. 5C). Highly damaged cells were noted in the 0.39 g/L treatment group after 4–18 h and in the highest concentration (0.52 g/L) after 4 and 8 h (tail DNA% 46–80, Fig. 5D). Along with extremely damaged cells appeared in 0.52 g/L treatment group after 18 h (tail DNA% > 80, Fig. 5E). The analysis of single cell gel electrophoresis results revealed that malathion could induce genotoxicity in roots and also showed a distinct concentration and time dependent relationship. Previous study suggested that oxidative stress could induce different kinds of negative effects including DNA damage, though excess ROS is a main reason of DNA damage^44^. The present study established the positive correlation of DNA damage and antioxidant enzyme activities with malathion concentration and duration of treatment indicating that oxidative DNA damage was the foremost reason of DNA lesion. These data manifested that malathion could affect not only on the normal physiological activity of cells but also damage DNA. Similar results were obtained in different organisms including fish^68^, Sprague-Dawley rats^69^ and human^70^.

It has been concluded that the exposure of malathion can induce cytotoxicity, phtotoxicity, oxidative stress and DNA damage in roots of *A. cepa* L. that may be one of the possible mechanism of malathion toxicity. Physiologically, malathion down-regulated the biosynthesis of sucrose. On contrary, the increased proline content elucidated that the malathion could also develop stress on plant roots. Molecular docking results showed that malathion could regulate antioxidant enzyme activities by interacting their residues. The biochemical analysis could be illustrated as tolerance mechanisms and may allow us to evolve strategies for alleviating the risk from pesticide contamination in crops.
